# Matrine Enhances the Pathogenicity of *Beauveria brongniartii* Against *Spodoptera litura* (Lepidoptera: Noctuidae)

**DOI:** 10.3389/fmicb.2019.01812

**Published:** 2019-08-13

**Authors:** Jianhui Wu, Xintong Yu, Xiaoshuang Wang, Liangde Tang, Shaukat Ali

**Affiliations:** ^1^Key Laboratory of Bio-Pesticide Innovation and Application, Engineering Research Centre of Biological Control, South China Agricultural University, Guangzhou, China; ^2^Key Laboratory of Integrated Pest Management on Tropical Crops, Ministry of Agriculture, Environment and Plant Protection Institute, Chinese Academy of Tropical Agricultural Sciences, Haikou, China

**Keywords:** natural chemicals, entomopathogenic fungi, matrine, synergism, *Spodoptera litura*

## Abstract

The repetitive application of pesticides at high doses against *Spodoptera litura* Fabricius has resulted in development of pesticide resistance and harmful effects to the natural environmental. Hence, finding alternate pest control strategies, such as entomopathogenic fungi or their application in combination with other natural chemicals, is of great importance to solve the abovementioned problems. This study presents the toxic effects of *Beauveria brongniartii* and matrine (individual or in combination with each other) against tobacco cutworm (*S. litura*). Different matrine treatments caused a dose dependent increase in *S. litura* mortality at different time intervals. The biological parameters of *B. brongniartii* (germination rate and average daily mycelia growth) were not inhibited by different matrine treatments. Different conidial concentrations of *B. brongniartii* caused significantly different mortalities of 2nd instar *S. litura* larvae at different time intervals. Different combined treatments of *B. brongniartii* and matrine showed a significant synergistic effect against *S. litura* under laboratory and semi-field conditions. The current findings showed a strong synergistic action for combined application of *B. brongniartii* and matrine against *S. litura.* Our results will provide baseline information on combined application of entomopathogenic fungi and natural chemicals in integrated pest management programs against *S. litura*.

## Introduction

*Spodoptera litura* (Fabricius) (Lepidoptera: Noctuidae) also known as tobacco cutworm is a major threat to majority of cropping systems because of its generalist herbivores behavior. Almost 389 plant species from 109 families have been documented as host plants of *S. litura* (Qing et al., 2006). The management of *S. litura* is mostly carried out through heavy pesticide which is causing many adverse effects to human health as well as to other components of our environment (Ishtiaq et al., 2012; Ahmad and Mehmood, 2015). Apart from this, the repetitive pesticide application is making *S. litura* resistant to these chemicals. Zhang et al. (2014) has reported the development of moderate to high insecticide resistance by *S. litura* against different pesticides such astubefenozide, indoxacarb, spinosid, and emamectin benzoate. Hence, finding alternate pest control strategies such as natural control agents is need of the hour to solve the above-mentioned problems (Revathi et al., 2014).

Matrine is a naturally occurring heterocyclic compound derived from quinolizidine which is isolated from the roots of *Sophora flavescens* and *Sophora alopecuroides* (Fabales: Fabaceae) (Mao and Henderson, 2007; Zhang et al., 2012; Cheng et al., 2018). Matrine is known to have a wide range of medicinal activities like anti-cancer, anti-inflammatory, antimicrobial, antiviral, antifibrotic, and immunoinhibitory, etc. (Hu et al., 2010; Chao et al., 2013; Sun et al., 2016). Matrine is one of the most used traditional medicines in China being used for the treatment of diseases such as cancer, viral hepatitis, colpitis, skin inflammation, etc. (Nakajyo et al., 1983; Zhang et al., 2009, 2012). Matrine is also a well-known botanical pesticide because of its broad spectrum insecticide activities, anti-plant virus activity and fungicidal activity as well as being friendly to the environment (Li et al., 2010; Zanardi et al., 2015; Ali et al., 2017). Recent studies have shown the use of matrine in an isolated form or in a mixture with other botanical, synthetic and microbial pesticides to control different insect pests (termites, whiteflies, aphids, leaf hoppers, caterpillars and mites), fungal and bacterial diseases and nematodes in china (Yang and Zhao, 2006; Liu et al., 2007; Mao and Henderson, 2007; Li et al., 2010; Ali et al., 2017). Matrine has been commercialized under different trade names; however, their insecticidal activities are lower than the popular insecticides introduced by international pesticide companies during the last few years (Cheng et al., 2018). Based on the good biological activity and wide range it will be interesting to develop a practical strategy for preparation of insecticidal formulations in which matrine can be used in mixture with other natural pest control agents.

Entomopathogenic fungi are promising agents in regulation populations of *S. litura* (Chaudhari et al., 2016; Nguyen et al., 2017; Namasivayam et al., 2018; Sahayaraj et al., 2018; Kaur et al., 2019; Yang et al., 2019). Fungi belonging to genus *Beauveria* (Hypocreales: Cordycipitaceae) are one of the most common insect pathogenic fungal species, which can act as an alternative to pesticides against *S. litura* (Borja et al., 2018; Dhar et al., 2019; Yang et al., 2019). *Beauveria* species are known to cause widespread epizootics of insect populations because of their saprophytic behavior (Leathers et al., 1993). *Beauveria bassiana* (Balsamo), *B. brongniartii* (Saccardo), and *B. amorpha* (von Hoehnel) are the most effective as well as highly used insect pathogenic species belonging to genus *Beauveria* (Gupta, 2002). *Beauveria brongniartii* (Sacc.) Petch is a promising fungal pathogen, which is virulent against different insect species (Wada et al., 2010, 2016; Fan et al., 2013; Liu et al., 2016; Soni et al., 2017). Liu et al. (2016) determined the toxicity of *B. brongniartii* strain NEAU30503 against larval instars of two pest species belonging to order Lepidopetra (*Xestia c-nigrum* Linnaeus and *Agrotis ypsilon* Rottenberg). Their results showed LC_50_ values of 7.28 × 10^7^ and 3.85 × 10^7^ conidia/ml against *X. nigrum* amd *A. ypsilon*, respectively. The higher environmental tolerance, longer persistence and higher infection ability makes *B. brongniartii* a potential biocontrol agent against *S. litura* management. *B. brongniartii* isolate SB010 was used in this study due to its considerably higher pathogenic activity against different insect species observed during our recent studies (Huang, 2019; Ou, 2019).

This study deliberates the possible synergistic action of matrine and *B. brongniartii* against *S. litura* in laboratory and semi field conditions as both of these agents possibly affects their host through same site of action. Matrine is known to target insect acetylcholine receptors, which in turn effects acetylcholinestrase production (Liu et al., 2007). *B. brongniartii* enters its insect hosts through the penetration of their cuticle unlike the other microbial pathogens (bacteria, or viruses) which needs ingestion to induce disease (Wang et al., 2004). *B. brongniartii* is known to secrete different biochemicals such as enzymes and secondary metabolites during cuticle degradation, colonization and proliferation of host hemocoel (Thomas and Read, 2007; Fan et al., 2013). Host death is often due to a combination of the action of a fungal toxin, the physical obstruction of blood circulation, nutrient depletion and the invasion of organs (Fan et al., 2013). *Beauveria* species produce a secondary metabolite named bassianolide, which can affect acetylcholine receptors of insect muscles, reducing the production of acetylcholinesterase (Xu et al., 2009). Initially, the effects of multiple doses of matrine on conidial germination and mycelial growth of *B. brongniartii* were studied. Then, multiple dose levels of matrine, *B. brongniartii*, and their combinations were tested against *S. litura* as the majority of previous synergistic studies of matrine with other control agents (Hwang et al., 2009; Li et al., 2010) have used one level of each component. We hope that our results will provide baseline information on combined application of entomopathogenic fungi and natural chemicals in integrated pest management programs against *S. litura.*

## Materials and Methods

### Insect Rearing

Semi synthetic artificial diet prepared by following David et al.(1975) was used to rear *S. litura* at 26 ± 2°C; 65 ± 5% relative humidity and16 h: 8 h (light: dark) photoperiod.

### Insecticide and Fungal Preparations

Matrine (purity 95%) was obtained from Guangdong New Scene Bioengineering Company, Yangjiang, China. A stock solution of matrine (1.0 mg/mL) was prepared by dissolving matrine powder in methanol. Lower concentrations were prepared through serial dilutions by using methanol as solvent.

*Beauveria brongniartii* isolate SB010 originally isolated from soil obtained from the repository of Key laboratory of biopesticides innovation and application of Guangdong Province, South China Agricultural University, Guangzhou, China was used during these studies. Fungal inoculum (1 × 10^9^ conidia/mL) for this study were prepared by following Ali et al. (2017). Lower conidial concentrations were prepared by serial dilutions with deionized water containing 0.02% Tween 80.

### Effect of Matrine on Conidial Germination, and Mycelia Growth of *B. brongniartii*

The effect of matrine on germination of *B. brongniartii* was studied by adding five different concentrations of matrine (0.05, 0.125, 0.25, 0.5, and 1.0 mg/L) to 100 mL sterilized sabouraud dextrose liquid culture medium (SDA) in 250 mL Erlenmeyer flasks whereas culture medium without matrine served as control. *B. brongniartii* was added to each treatment and control flasks to final concentrations of 1 × 10^4^ conidia/mL. The experimental setup was incubated in a rotary shaker at 180 rpm and 26 ± 2°C for 3 days. The number of germinated conidia was observed every 24 h and transformed to percent germination. Fungal germlings having germ tube lengths longer than spore diameter were counted as germinated. The whole experiment was repeated thrice with fresh conidial suspension prepared every time.

The influence of matrine on radial growth of *B. brongniartii* was observed by layering five different concentrations of matrine (0.05, 0.125, 0.25, 0.5, and 1.0 mg/mL) on solidified PDA disks (PDA medium (10 mL) was poured into petri dishes (9 cm) and left to solidify for 2 h) and left to dry overnight. PDA plates without matrine layer served as control. Mycelial disks of *B. brongniartii* (2 cm diameter) were inoculated to different treatments and control Petri plates. The whole experimental setup was incubated at 25 ± 2°C, 80 ± 5% relative humidity and 16 L: 8 D photoperiod. The colony diameters were measured by using the method of Ali et al. (2009) on daily basis until 7 days.

### Efficacy of *B. brongniartii* Against *S. litura*

Five different conidial concentrations (1 × 10^5^, 1 × 10^6^, 1 × 10^7^, 1 × 10^8^_,_ and 1 × 10^9^ conidial/mL) were added to the molten state of artificial diet prepared by following David et al. (1975). Artificial diet without the addition of fungal conidia served as control. Freshly molted 2nd instar *S. litura* larvae were individually placed in plastic cups (4 cm diameter) to feed on 1 g of pre-treated as well as control diet. The cups were incubated at 25 ± 2°C, 80 ± 5% relative humidity and 16 L:8 D h photoperiod. There were 20 larvae used for each treatment and the whole experimental setup was repeated thrice. Larval mortality was recorded on a daily basis for 7 days. Fungal infection of larvae was identified by the darker body color and, later on, by outgrowth of mycelia from larval bodies.

### Efficacy of Matrine Against *S. litura*

Five different matrine concentrations (0.05, 0.125, 0.25, 0.5, and 1.0 mg/L) were added to the molten state of artificial diet. Artificial diet without the addition of matrine served as control. Freshly molted 2nd instar *S. litura* larvae were individually placed in plastic cups (4 cm diameter) to feed on one gram of pre-treated as well as control diet. There were 20 larvae used for each treatment and the whole experimental setup was repeated thrice. The whole experimental setup was incubated at 25 ± 2°C, 80 ± 5% relative humidity and 16L: 8D h photoperiod and larval mortality was recorded on daily basis for 7 days.

### Toxicity of *B. brongniartii* and Matrine Alone or in Combination With Each Other Against *S. litura* Under Laboratory Conditions

Individual or joint treatments of *B. brongniartii* and matrine as shown in Table 1 were added to the molten state of artificial diet. Artificial diet without the addition of fungal conidia served as control. Freshly molted 2nd instar *S. litura* larvae were individually placed in plastic cups (4 cm diameter) to feed on 1 g of pre-treated as well as control diet. The experimental setup was incubated at 25 ± 2°C, 80 ± 5% relative humidity and 16 L: 8 D h photoperiod. The mortality was observed as described in above section.

**TABLE 1:** Combinations of *Beauveria brongniartii* and matrine tested in efficacy studies.

**Treatment**	***Beauveria brongniartii*(conidia/mL)**	**Matrine(mg/L)**
T0	0	0
T1	0	0.5
T2	0	1.0
T3	1 × 10^8^	0
T4	1 × 10^9^	0
T5	1 × 10^8^	0.5
T6	1 × 10^8^	1.0
T7	1 × 10^9^	0.5
T8	1 × 10^9^	1.0

### Toxicity of *B. brongniartii* and Matrine Alone or in Combination With Each Other Against *S. litura* Under Greenhouse Conditions

The efficacy of Individual or joint treatments of *B. brongniartii* and matrine (Table 1) against 2nd instar *S. litura* larvae were also tested under greenhouse conditions by following the method of Xu et al. (2011) with some modifications. Freshly molted 2nd instar *S. litura* larvae were placed on cabbage leaves. Ten larvae were placed on each leaf and three leaves were selected from each plant making a total of 30 larvae per plant. Different treatments were sprayed on leaves with 500 mL hand sprayer following Vidal et al. (1998). The treated leaves were covered with plastic screen to avoid the escape of insects. The leaves of control groups were sprayed with 0.02% Tween 80. The entire experiment was repeated three times at different dates. Larval mortality was recorded on a daily basis for 7 days.

### Data Analysis

Percent of germination and radial growth of *B. brongniartii* was analyzed through one-way ANOVA and means were separated by Tukey’s HSD test (Tukey’s < 0.05). Mortality values of *S. litura* in response to different concentrations of *B. brongniartii* and matrine at different time intervals were arcsine square-root transformed followed by two-way ANOVA and means were separated by Tukey’s HSD test (Tukey’s < 0.05), when *F*-value was significant. All data analysis was performed by using SAS 8.1 software (SAS Institute, 2000).

The presence of possible synergism between *B. brongniartii* and matrine was calculated by following Xu et al. (2011). Initially, the corrected mortality over control was calculated by following equation (Abbott, 1925).

$$\text{Corrected mortality}\,(M)=\frac{\left(\text{Mortality in response to treatment}-\text{Mortality in response to control}\right)}{\left(1-\text{Mortality in response to control}\right)}$$

The expected mortality in response to different combined treatments of *B. brongniartii* and matrine as well as chi square values to determine the kind of interaction were calculated through following equations

$$\mathrm{Expected}\,\mathrm{mortality}\,\left(M_{\text{e}}\right)=M_{\text{A}}+M_{\text{B}}\times \left(1-M_{\text{A}}\right)$$

where *M*_e_ is the expected mortality for additive mortality; *M*_A_, *M*_B_ and *M*_AB_ are the observed mortalities for *B. brongniartii*, matrine and their combination, respectively. The significant differences between the observed and expected mortality were defined through significance of the chi square values calculated through following equation

$$\mathrm{Chi}\,\mathrm{square}\,\left(\chi^{2}\right)=\frac{\left(M_{\text{AB}}-M_{\text{e}}\right)\times 100\times \left(M_{\text{AB}}-M_{\text{e}}\right)}{M_{\text{e}}}$$

Then *P*-values were looked up in chi square table for df = 1.

If the *M*_AB_ was significantly lower than *M*_e_ (when the calculated χ^2^ was lower than expected χ^2^ value observed from chi square table), it meant antagonism. If the *M*_AB_ was significantly higher than *M*_e_ (when the calculated χ^2^ was higher than expected χ^2^ value observed from chi square table), it meant synergism. Otherwise the mortality was additive (Xu et al., 2011).

## Results

### Influence of Matrine on Conidial Germination and Radial Growth of *B. brongniartii*

**FIGURE 1:**
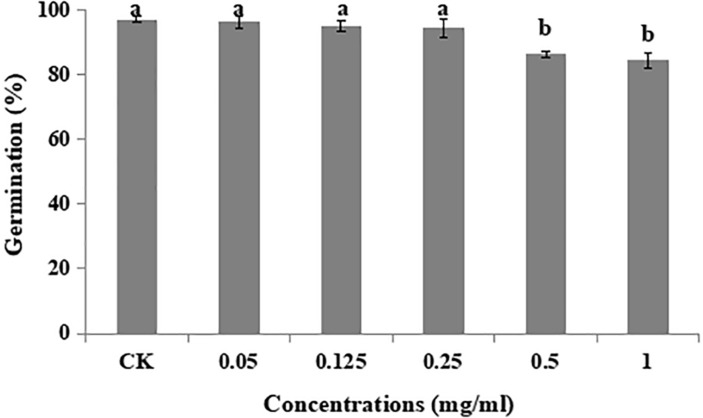
Effect of matrine on percent germination of *Beauveria brongniartii.* Bars having different letters are significantly different from each other (Tukey’s *P* < 0.05).

Different concentrations of matrine had a non-significant effect on germination rate (%) of *B. brongniartii* when compared with control. The highest rate of germination (97 ± 0.086%) was observed for control whereas the lowest was observed for matrine concentration of 1.00 mg/L with mean value of 84 ± 3.37% (Figure 1).

**FIGURE 2:**
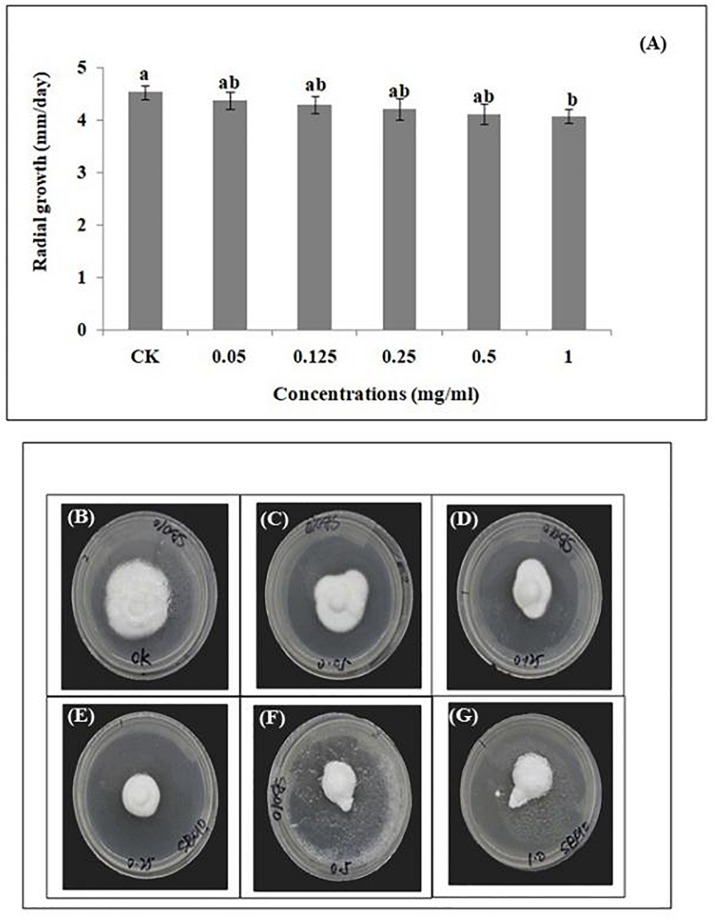
Effect of matrine on radial growth of *Beauveria brongniartii.* **(A)** Radial growth (mm/day) in response to different matrine concentrations; **(B–G)** Growth of *Beauveria brongniartii* on different PDA plates having different matrine concentrations. Bars having different letters are significantly different from each other (Tukey’s *P* < 0.05).

The average daily mycelial growth of *B. brongniartii* was not significantly affected by different matrine concentrations (except 1.0 mg/ml) when compared with control (*F* = 45.72; df = 5.12; *P* = 0.049). The highest average daily radial growth (4.53 ± 0.13 mm/day) was observed in the control whereas the lowest germination was observed for matrine concentration of 1.00 mg/L with a mean value of 4.13 ± 0.08 mm/day (Figure 2).

### Efficacy of *B. brongniartii* Against *S. litura*

**FIGURE 3:**
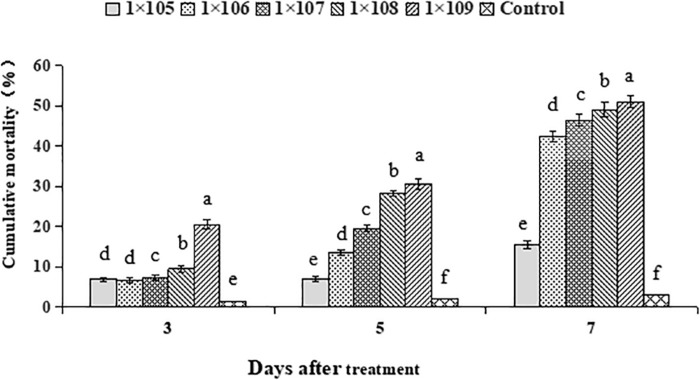
Percentage mortality of *Spodoptera litura* in response to different *Beauveria brongniartii* concentrations. Bars having different letters are significantly different from each other (Tukey’s *P* < 0.05).

Average cumulative mortality of 2nd instar *S. litura* larvae in response to *B. brongniartii* differed significantly at different time intervals (*F*_2__,__28_ = 33.68, *P* < 0.01). Average cumulative mortality of 2nd instar *S. litura* larvae at different time intervals differed significantly among different concentrations of *B. brongniartii* (*F*_4__,__28_ = 68.95, *P* < 0.01). The interaction effect between different time intervals and different *B. brongniartii* concentrations for mortality of 2nd instar *S. litura* larvae was also significant statistically (*F*_8__,__28_ = 25.61, *P* < 0.01). The highest mortality rates after 3, 5, and 7 days after fungal treatment were observed for conidial concentration of 1 × 10^9^ conidia/mL; whereas lowest mortalities were observed for conidial concentration of 1 × 10^5^ conidia/mL (Figure 3). Based on the above mortality data, the concentration-mortality response regression analysis for *B. brongniartii* was carried out to calculate the medial lethal concentration (LC_50_). The LC_50_ value of *B. brongniartii* against *S. litura* was 1.4 × 10^8^ conidia/mL after 7 days of fungal application.

### Efficacy of Matrine Against *S. litura*

**FIGURE 4:**
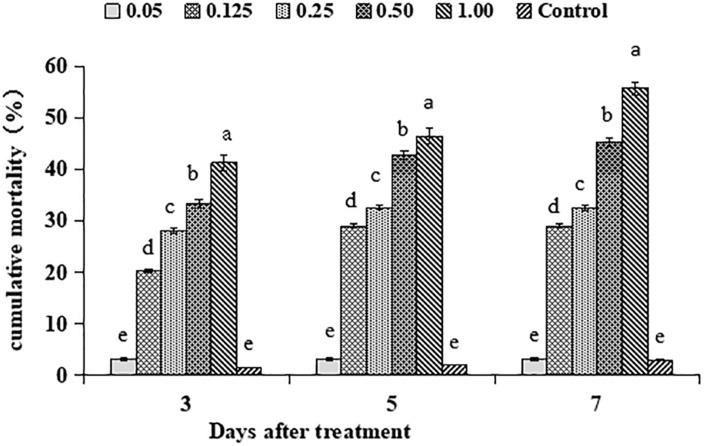
Percentage mortality of *Spodoptera litura* in response to different matrine concentrations. Bars having different letters are significantly different from each other (Tukey’s *P* < 0.05).

The main effect of different matrine concentrations on mean cumulative adjusted mortality for 2nd larval instar of *S. litura* was significant (*F*_4__,__28_ = 48.80, *P* < 0.01). The time duration after matrine application also showed a significant effect on mean cumulative adjusted mortality for 2nd larval instar of *S. litura* (*F*_2__,__28_ = 29.36, *P* < 0.01). Similarly, there was a significant interaction effect on *S. litura* mortality involving matrine concentrations and time duration (*F*_8__,__28_ = 31.91, *P* < 0.01). The highest mortality rates after 3, 5, and 7 days post treatment were observed for concentration of 1.00 mg/L whereas, lowest mortalities were observed for conidial concentration of 0.05 mg/L (Figure 4). Based on the above mortality data, the concentration-mortality response regression analysis for matrine was carried out to calculate the medial lethal concentration (LC_50_). The LC_50_ value of matrine against *S. litura* was 0.80 mg/L.

### Efficacy of *B. brongniartii* and Matrine Alone or in Combination With Each Other Against *S. litura* Under Laboratory Conditions

**FIGURE 5:**
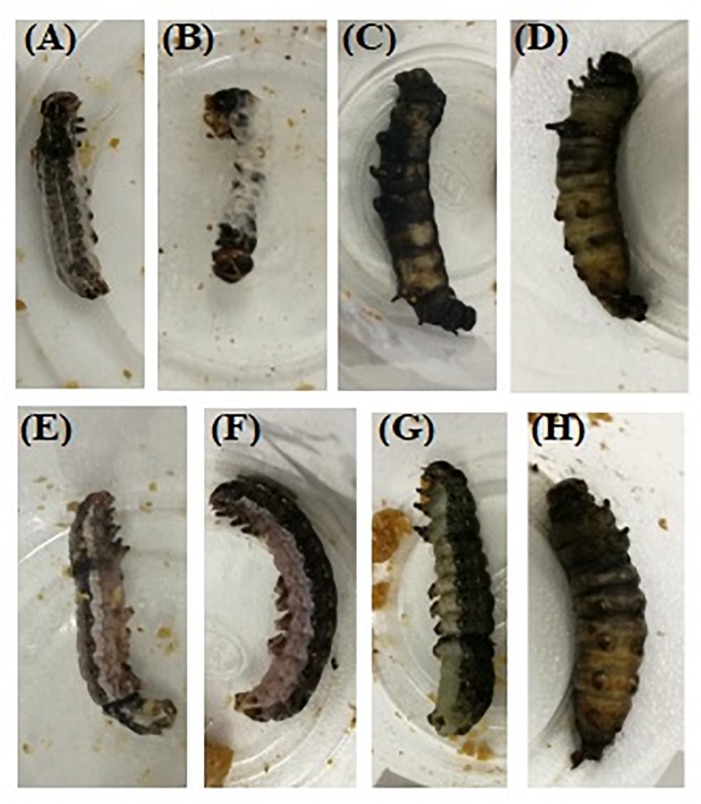
Mortality symptoms of *S. litura* after 5 days of treatment with *Beauveria brongniartii* and matrine alone or in combination with each other **(A)** *B. brongniartii* 1 × 10^8^ conidia/mL; **(B)** *B. brongniartii* 1 × 10^9^ conidia/mL; **(C)** Matrine 0.5 mg/mL; **(D)** Matrine 1.0 mg/mL; **(E)** *B. brongniartii* 1 × 10^8^ conidia/mL + Matrine 0.5 mg/mL; **(F)** *B. brongniartii* 1 × 10^8^ conidia/mL + Matrine 1.0 mg/mL; **(G)** *B. brongniartii* 1 × 10^9^ conidia/mL + Matrine 0.5 mg/mL, and **(H)** *B. brongniartii* 1 × 10^9^ conidia/mL + Matrine 1.0 mg/mL.

The different *B. brongniartii* and matrine joint treatment showed combinations significantly higher mortalities of 2nd instar *S. litura* larvae when compared with control as well as individual treatments of *B. brongniartii* or matrine (*F*_7__,__48_ = 39.91, *P* < 0.01). The *S. litura* mortality in response to different *B. brongniartii* and matrine treatments (individual or joint treatments) under laboratory conditions differed significantly at different time intervals (*F*_2__,__48_ = 36.21, *P* < 0.01) as did the interaction effect of different treatments and time intervals (*F*_14__,__48_ = 78.46, *P* < 0.01). Moreover, joint treatments of *B. brongniartii* and matrine had a significant synergistic interaction at different time interval (3, 5, and 7 days) post treatment as the mortality values observed for combined treatments were significantly higher than expected mortality values (Table 2). The mortality symptoms of *S. litura* treated with *B. brongniartii* and matrine alone or in combination with each other are shown in Figure 5. The highest *S. litura* mortality (100%) was observed for T8 after 7 days of treatment whereas lowest *S. litura* mortality (9.43%) was observed for T3 after 3 days of fungal application. The rates of mortality observed for T6, T7, and T8 were statistically at par after 7 days of treatment (Table 2).

**TABLE 2:** Mean percent adjusted mortality of instar *Spodoptera litura* on mixtures of *Beauveria brongniartii* and matrine under laboratory conditions.

**Treatments**	**Mean percent adjusted mortality at different intervals**		
	**3 days**	**5 days**	**7 days**
T0	0.010.00*p*	0.010.00*p*	0.030.00*p*
T1	33.330.91*m*	42.720.76*kl*	45.300.84*jk*
T2	41.241.56*l*	46.391.58*jk*	55.671.25*g*
T3	9.430.97*o*	23.380.75*n*	49.061.75*ij*
T4	20.411.14*n*	30.611.35*m*	51.021.48*hi*
T5	54.69^*^0.91*gh*(39.62,5.22)	76.62^*^2.04*d*(58.9,4.94)	91.64^*^1.29*b*(72.14,5.27)
T6	61.71^*^1.23*f*(46.78,4.32)	79.48^*^2.03*d*(61.56,4.94)	96.52^*^1.41*a*(77.42,4.72)
T7	61.23^*^1.77*f*(46.94,4.21)	79.71^*^3.1*d*(60.25,5.83)	96.74^*^2.83*a*(73.21,7.56)
T8	68.01^*^2.62*e*(53.23,4.09)	84.80^*^4.21*c*(62.80,7.15)	100a^*^(78.29,6.02)

For treatment details see Table 1. Means (M ± SE) followed by different letters are significantly different (Tukey’s *P* < 0.05). Data in brackets shows the expected mortality and the chi-square value, respectively. ^*^ represent the combined treatment having synergistic interaction through data analysis.

### Efficacy of *B. brongniartii* and Matrine Alone or in Combination With Each Other Against *S. litura* Under Semi-Field Conditions

The *S. litura* mortality in response to different *B. brongniartii* and matrine treatments (individual or joint treatments) under semi-field conditions differed significantly at different time intervals (*F*_2__,__48_ = 51.77, *P* < 0.01). The different *B. brongniartii* and matrine joint treatment showed combinations significantly higher mortalities of 2nd instar *S. litura* larvae when compared with the control as well as individual treatments of *B. brongniartii* or matrine (*F*_7__,__48_ = 47.08, *P* < 0.01). Similarly there was a significant interaction effect on *S. litura* mortality involving different treatment and time intervals (*F*_14__,__48_ = 42.57, *P* < 0.01). Moreover, joint treatments of *B. brongniartii* and matrine showed a significant synergistic interaction at different time intervals (3, 5, and 7 days) post treatment as the mortality values observed for combined treatments were significantly higher than expected mortality values (Table 2). The highest *S. litura* mortality (89.25%) was observed for T8 after 7 days of treatment whereas lowest *S. litura* mortality (6.67%) was observed for T3 after 3 days of fungal application. The rates of mortality observed for T7 and T8 were statistically at par after 7 days of treatment (Table 3).

**TABLE 3:** matrine under field conditions.

**Treatments**	**Mean percent adjusted mortality at different intervals**		
	**3 days**	**5 days**	**7 days**
T0	0.100.00*p*	0.100.00*p*	0.0.200.00*p*
T1	27.040.87*m*	39.120.91*k*	44.231.02*j*
T2	37.511.23*k*	43.571.39*j*	52.381.39*i*
T3	6.670.94*o*	16.672.53*n*	30.002.30*lm*
T4	16.671.63*n*	30.002.22*lm*	31.671.79*l*
T5	50.00^*^4.16*i*(31.8,6.31)	67.00^*^5.2*f*(49.25,5.39)	79.52^*^4.72*c*(62.13,4.58)
T6	59.33^*^5.88*h*(41.66,7.20)	71.00^*^4.05*e*(53.08,5.04)	87.67^*^4.79*a*(66.61,6.24)
T7	62.3^*^3.74*gh*(39.16,6.59)	75.19^*^0.52*d*(57.37,5.41)	83.51^*^4.70*b*(62.99,6.35)
T8	65.303.17*fg*(47.91,6.08)	77.055.83*cd*(60.59,4.44)	89.25^*^4.95*a*(67.37,6.94)

For treatment details see Table 1. Means (M ± SE) followed by different letters are significantly different (Tukey’s *P* < 0.05). Data in brackets shows the expected mortality and the chi-square value, respectively. ^*^ represent the combined treatment having synergistic interaction through data analysis.

## Discussion

Previous studies on *B. brongniartii* have only explained the pathogenic ability as well as lethal and sub lethal effects of this fungi against different insect pests (Fan et al., 2013; Goble et al., 2015; Mayerhofer et al., 2015; Soni et al., 2017). However, very few studies have elaborated the possible compatibility of *B. brongniartii* with natural chemicals (derived from plant or any other living organism) and other chemicals with novel mode of action (Sushil et al., 2018) .This study reports the synergistic interaction of *B. brongniartii* with matrine (plant derived chemical) against *S. litura* under laboratory as well as semi field conditions.

Different concentrations of matrine were tested for their influence on the radial growth and conidial germination rates of *B. brongniartii*. The results showed a low inhibition of radial growth and percent conidial germination. These results are similar to the findings of Xu et al. (2011) who observed similar effects of botanical extract 20-Hydroxyecdysone (derived from the plant, *Ajuga nipponensis* Makino) on growth and conidial germination of entomopathogens *Isaria fumosorosea.*

The main reason behind testing the efficacy of different conidial concentrations was to observe the pathogenic potential of *B. brongniartii* against 2nd instar larvae of *S. litura* and to find out the optimum conidial concentration of *B. brongniartii* to be used in subsequent experiments. Our results showed that 2nd instar larvae of *S. litura* were susceptible to *B. brongniartii* having a median concentration value of 1.4 × 10^8^ conidia/mL after 7 days of fungal application. These results are different from the findings of Liu et al. (2016) who observed LC_50_ values of 7.28 × 10^7^ and 3.85 × 10^7^ conidia/ml against *X. nigrum* and *A. ypsilon*, respectively. Our findings are also different from Dhar et al. (2019) who observed LC_50_ value of 9.35 × 10^4^ conidia/mL for *B. bassiana* isolate BbR2 against *S. litura* after 7 days of fungal application. Different matrine concentrations were tested for their toxicity against the population of *S. litura* reared at Key laboratory of biopesticides innovation and application of Guangdong Province, South China Agricultural University, Guangzhou, China under standard laboratory conditions. Based on the above mortality data, the medial lethal concentration (LC_50_) of matrine against *S. litura* was 0.80 mg/L after 7 days of application. The LC_50_ of matrine against *S. litura* observed are slightly higher than the LC_50_ values of matrine against *S. litura* (0.45 ml/L) observed by Han et al. (2015). The variation in LC_50_ values may be explained by the changes in insect populations or difference in ecological niche of the tested insect populations. Our results are similar to the findings of Ali et al. (2017) who observed LC50 value of 0.83 mg/L against *Bemisia tabaci* Gennadius.

The interaction of entomopathogenic fungi with plant derived chemicals have been assessed through different mathematical models (Finney, 1971; Tabashnik, 1992).However, selection of earlier used models might not be feasible for this study as mortality of mixture comes from two sources (Xu et al., 2011) as *B. brongniartii* and matrine are known to target insect acetylcholine (Ach) receptors ultimately killing the insect through failure in acetylcholinesterase production (Liu et al., 2007; Xu et al., 2009). Joint application of *B. brongniartii* and matrine against *S. litura* caused a significant increase in mortality values under laboratory as well as field conditions, showing a strong synergistic interaction. The level of synergism shown by this study are consistent with Ali et al. (2017) who observed similar increase in mortalities of *B. tabaci* treated with different combinations of matrine and *Lecanicillium muscarium*. The level of synergism observed under semi-field conditions was slightly lower than the laboratory conditions which can be explained by the variations in different biotic and abiotic factors or behavior of the target pest under semi field conditions (Xu et al., 2011). Our results are also similar to the studies of Han et al. (2015). Their results demonstrated significantly higher *S. exigua* mortality for the mixtures of botanicals (matrine or neem) with Bacillus thuringiensis when compared with control and only botanicals (matrine or neem) treatments.

## Conclusion

Our findings reports promising results for combined application of *B. brongniartii* and matrine in controlled as well as field conditions. These results will provide baseline information for the development as well as utilization of botanical + microbial insecticide based formulations in *S. litura* management programs. However, further studies are required for the detailed elaboration of sublethal effects of the matrine and *B. brongniartii* on growth and development of *S. litura* as well as the physiological processes involved in the said synergistic effect.

## Data Availability

The raw data supporting the conclusions of this manuscript will be made available by the authors, without undue reservation, to any qualified researcher.

## Author Contributions

JW and SA conceived and designed the research. XY and XW conducted the experiments. LT and SA analyzed the data. SA wrote the manuscript. All authors read and approved the manuscript.

## Conflict of Interest Statement

The authors declare that the research was conducted in the absence of any commercial or financial relationships that could be construed as a potential conflict of interest.
